# Diversity, distribution and conservation of crocodiles (Order: Crocodylia) in Guinea-Bissau, West Africa

**DOI:** 10.1038/s41598-025-08789-3

**Published:** 2025-07-09

**Authors:** Cristian Pizzigalli, Aissa Regalla, Ana Filipa Palmeirim, Luís Palma, Manuel Lopes-Lima, Orly Razgour, Raquel Godinho, William Alexandre Intipe, José Carlos Brito

**Affiliations:** 1 CIBIO, Centro de Investigação em Biodiversidade e Recursos Genéticos, Campus de Vairão, Universidade do Porto, Vairão, 4485-661 Portugal; 2 BIOPOLIS Program in Genomics, Biodiversity and Land Planning, CIBIO, Campus de Vairão, Vairão, 4485- 661 Portugal; 3 Departamento de Biologia da Faculdade de Ciências, Universidade do Porto, Porto, Portugal; 4 Biosciences, Faculty of Health and Life Sciences, University of Exeter, Exeter, UK; 5 Instituto da Biodiversidade e das Áreas Protegidas, Dr. Alfredo Simão da Silva, Av. D. Settimio Arturo Ferrazzetta, Bissau, Guinea; 6 Laboratório de Ecologia e Zoologia de Vertebrados, Instituto de Ciências Biológicas, Universidade Federal do Pará, Belém, Brasil; 7 CIBIO, Centro de Investigação em Biodiversidade e Recursos Genéticos, Instituto Superior de Agronomia, Universidade de Lisboa, Lisboa, 1349-017 Portugal; 8 Department of Zoology, Faculty of Sciences, University of Johannesburg, Auckland Park 2006, Johannesburg, South Africa

**Keywords:** West Africa, Non-invasive genetic, Habitat degradation, Human-wildlife conflict, Freshwater biodiversity, *Crocodylus niloticus*, Biodiversity, Taxonomy, Herpetology, Biodiversity, Biogeography, Conservation biology, Freshwater ecology

## Abstract

Challenges in freshwater organism conservation in West Africa are worsened by significant knowledge gaps, even for charismatic species like crocodiles. This study addresses these gaps by assessing crocodile diversity, distribution, and conservation threats in Guinea-Bissau, where existing data is outdated. We used visual surveys, inquiries, molecular barcoding, camera trapping, and bibliographic reviews to investigate crocodile populations. Notably, we found evidence suggesting the Nile crocodile (*Crocodylus niloticus*), previously thought extinct in West Africa since about 200 years, may persist in Guinea-Bissau’s Cacheu region. We also confirmed the presence of the West African crocodile (*Crocodylus suchus*) in major river basins and coastal lagoons, including the Bijagós Archipelago, and the West African dwarf crocodile (*Osteolaemus cf. tetraspis*) in the southern mainland and the Bijagós Archipelago. Habitat loss and deliberate killings were identified as major threats. Standardized surveys and genetic sampling are essential to assess population size, connectivity, and genetic diversity, informing evolutionary studies and conservation planning. Conservation efforts should prioritize habitat protection through community-managed reserves and restoration initiatives. Additionally, engaging local communities to raise awareness and develop conflict mitigation strategies is crucial, particularly in areas with human-crocodile interactions.

**Supplementary Information:**

The online version contains supplementary material available at 10.1038/s41598-025-08789-3.

## Introduction

Freshwater organisms are among the most threatened biodiversity on the planet^1^ with major threats including pollution, dam construction, water extraction, land use change, agriculture, invasive species and disease^2^. Of these threats, agriculture and logging are the ones mostly affecting freshwater tetrapods through habitat degradation and loss^2^. This conservation crisis is exacerbated by the lack of knowledge on diversity and distribution of African wildlife, leading to a silent biodiversity decline^3^. West Africa is recognised as a global biodiversity hotspot^4–7^. However, its biodiversity is still deeply understudied when compared to other areas of the planet, with most of the published information focused on the southern countries of the region and skewed towards few taxonomic groups (i.e. mammals, birds and amphibians)^3,8^. Gaps on the diversity and distribution of organisms (Linnean and Wallacean shortfall), have deep impact in the understanding of biodiversity patterns and evolution, affecting also the efficient implementation of conservation policies^9^ even for large and charismatic species such as crocodiles.

Crocodilians are among the most charismatic aquatic tetrapods, playing a crucial role in their ecosystems as ecosystem engineers, apex predators and bioindicators^10–15^ being also culturally important for traditional medicine and spiritual beliefs^16^. African crocodilians are primarily found in freshwater rivers, lakes, and marshes, although some species can tolerate brackish or even saltwater. In the last 15 years, the description of the genus *Mecistops*, the resurrection of the West African crocodile (*Crocodylus suchus*, Geoffroy Saint-Hilaire, 1807), and of the Central African slender-snouted crocodile (*Mecistops leptorhynchus*, Bennett 1835)^17–19^ demonstrated that much remains to be discovered about the diversity, ecology and distribution of African crocodiles. Three crocodile species are known to occur in West Africa to date:

1) *Crocodylus suchus* is the only species of the genus *Crocodylus* recognised to be present in West Africa^17,20–22^. *Crocodylus suchus* can grow up to four metres, but most individuals are between 2 and 3.3 m long. The smaller size and non-aggressive behaviour towards humans seem to be the only phenotypic features distinguishing this species from the larger and more aggressive *Crocodylus niloticus* (Laurenti, 1768), which grows on average to more than 5 m long^17^. *Crocodylus suchus* current global distribution ranges from Mauritania to the Central African Republic, and as far east as Uganda. Although more than 10 years have now passed since its resurrection as a species, the IUCN Red List assessment for *C. suchus* is still pending, as well as its validation as a full species and its assessment by the Convention of International Trade of Endangered Species (CITES) legislation^23^. Like other crocodilians in West Africa, this species is likely threatened by increasing anthropisation and overexploitation of natural resources, and it is traded for meat and skin in Sub-Saharan Africa^24^.

2) *Mecistops cataphractus* (Cuvier, 1825). The genus *Mecistops* was until recently considered to be monospecific, but it now comprises two species: *M. cataphractus* and *M. leptorhynchus*^18,19^. Although the historical distribution of *M. cataphractus* likely ranged from The Gambia to the Cameroon volcanic line^18^ the species may now be restricted to the Republic of Guinea, Ivory Coast, and Ghana, with a population persisting in the Gambia River^19^. The species is considered as Critically Endangered by the IUCN Red List, and its last assessment predates the formal description of *M. leptorhynchus*, therefore the Red List still considers the two *Mecistops* species as one^25^. The taxonomy of *M. cataphractus* also needs to be updated in CITES (listed as *Crocodylus cataphractus* in Appendix I^23^S). Current threats to the species include small-scale subsistence fisheries, which contributes to reduced availability of prey and incidental mortality in fishing nets, and habitat degradation due to the land conversion from forest to commodity plantations or settlements^19,25^.

3) The most western lineage of the genus *Osteolaemus*, which has a distinct evolutionary history and geographic distribution from the two congeneric recognised species, *Osteolaemus tetraspis* Cope, 1861 and *Osteolaemus osborni* (Schmidt, 1919)^26^. However, since the validity of this candidate new species had not been tested yet, and an updated taxonomic revision of the genus is still pending, we will refer to it as *Osteolaemus cf. tetraspis*. The distribution of the *O. cf. tetraspis* lineage has been considered to extend from The Gambia to at least Ghana^26–28^. The only IUCN Red List assessment of the genus *Osteolaemus* dates back to 1996 and considers the genus to be monospecific and listed as Vulnerable^29^. However, among the three lineages of the genus, *O. cf. tetraspis* is likely to be the most threatened and with a higher risk of extinction^30^. *Osteolaemus cf. tetraspis* is considered a valuable commodity along its distribution, and its non-aggressive nature and small size make it the most heavily hunted crocodile in West and Central Africa^15,31,32^. The CITES also recognises the genus to be monospecific (*O. tetraspis*), with all populations listed in Appendix I^23^. Hunting and human-driven habitat transformation are likely the major threats to the persistence of this taxon^30,31^.

Although crocodiles are charismatic and economically valuable keystone species, the information available on their diversity, distribution and threats in Guinea-Bissau is mostly outdated and incomplete. Recent studies confirm that *Crocodylus suchus* is widespread across the country, while *O. cf. tetraspis* is mostly found in the south, with some scattered populations in the Cacheu region. Both species were also present in the Bijagós Archipelago, at least until 2012^27,33^. The presence of *M. cataphractus* in the country has long been debated and it has been suggested that it could inhabit the coastal lagoons of Guinea-Bissau and the major watercourses in the bordering regions with Senegal and the Republic of Guinea^27^.

Additionally, two large (> 3.5 m total length) crocodiles appear to be responsible for the killing of three people in the Cacheu region in 2015^34^. Crocodile attacks on humans are not new to Guinea-Bissau, although previous records were limited to the Bijagós Archipelago^33,35^. A body size greater than 3.5 m and aggressive behaviour are not expected in *C. suchus* (Geoffroy Saint-Hilaire, 1807)^17^ raising the possibility that relict populations of the Nile crocodile (*Crocodylus niloticus*) may persist in the country. *Crocodylus niloticus* was present in West Africa in the Senegal River until at least 1803^17^. However, the species is now known to be extinct in the region and distributed only in eastern, central and southern Africa^22,36^.

This study aims to provide updated information on the diversity, distribution, and conservation of crocodiles in Guinea-Bissau, by answering the following questions: (1) How many extant species of crocodiles occur in Guinea-Bissau? (2) How are these species spatially distributed? (3) What are the major threats to the persistence of crocodiles in the country? Using a combination of visual surveys, inquiries, molecular data, opportunistic bycatch images from camera trapping, and bibliographic review of the available information, we aim to provide new insights into how crocodile diversity is distributed across the country. Finally, by classifing threats following the guidelines established by the IUCN Red List Unified Classification of Direct Threats, we also aim to gather comprehensive information on the major threats facing these species in Guinea-Bissau.

## Results

### Species diversity

We successfully sequenced 52 of the 69 samples collected (Table S1). The probability of assignment (PI) of our query sequences to the NCBI dataset ranged from 96 to 100%, with the first five sequences selected by BLAST as more similar to our query sequences. Query cover was always 100%. BLAST results highlighted the presence of three species among our sequences: Nile crocodile (*Crocodylus niloticus*), West African crocodile (*Crocodylus suchus*), and the West African dwarf crocodile (*Osteolaemus* cf. *tetraspis*). When blasting our query sequences of *C. suchus*, among the 10 sequences selected by BLAST as the most similar to our query sequence, we had matching sequences labelled as *C. niloticus*, although they had already been identified as belonging to *C. suchus* in previous studies^17,37^.

**Fig. 1:**
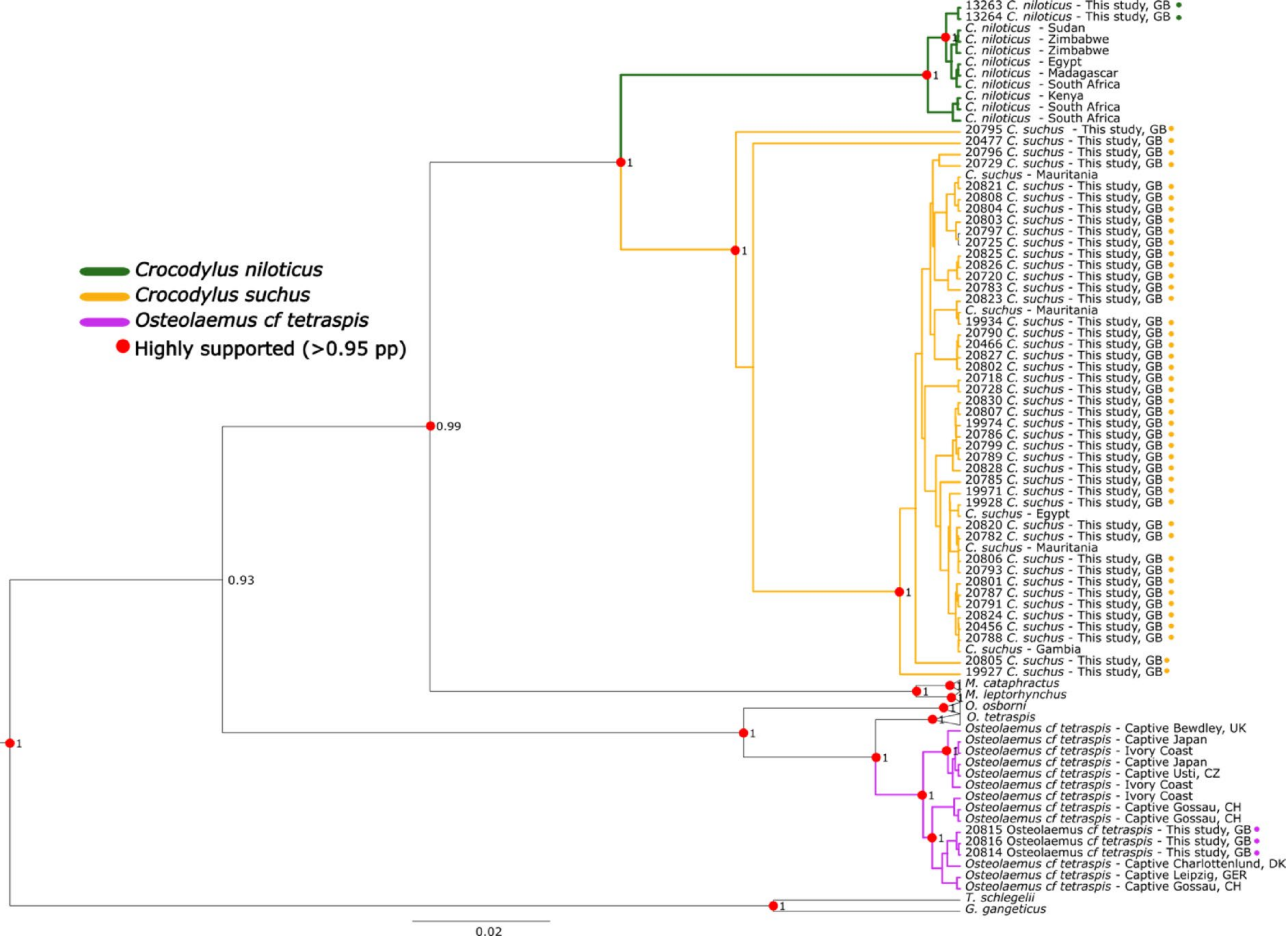
Placement of study samples within the African crocodilian phylogenetic tree. Colours of the branches refer to the species found in Guinea-Bissau in this study, namely: the Nile crocodile (*C. niloticus* in green), the West African crocodile (*C. suchus* in yellow), and the West African dwarf crocodile (*O. cf. tetraspis* in purple). Red dots at the nodes represent highly supported nodes (> 95% pp), coloured dots next to the branch description represent the samples collected for this study. The red rectangle in the inset map of Africa represents the geographic location of Guinea-Bissau. This figure and maps within it, were produced using QGIS version 3.36 (QGIS Development Team, 2024; https://qgis.org).

All species included in our analyses formed highly supported (> 99% pp) monophyletic lineages, except for the split between the *Osteolaemus* species complex and the cluster containing the genera *Crocodylus* and *Mecistops* (93% pp) (Fig. 1). The overall topology of our phylogenetic tree resembles the topology of the tree constructed by previous studies^17^. The results of our phylogenetic analyses clearly cluster two of our sequences with published sequences of *C. niloticus* from eastern and southern Africa, 44 sequences with published sequences of *C. suchus*, and three sequences with published sequences of *O. cf. tetraspis* corresponding to the possible new, undescribed species. Of the 44 sequences clustering with *C. suchus* one (20795) from a sample collected in the eastern sector of the Corubal River considerably diverged within the species cluster with high support (100% pp).

During our field surveys we found no evidence of *M. cataphractus*. However, local hunters and fishermen repeatedly pointed out the presence of another “type” of crocodile, which was meticulously described as having the appearance of *C. suchus*, but with a brownish colouration, and a longer and thinner head.

### Distribution

**Fig. 2:**
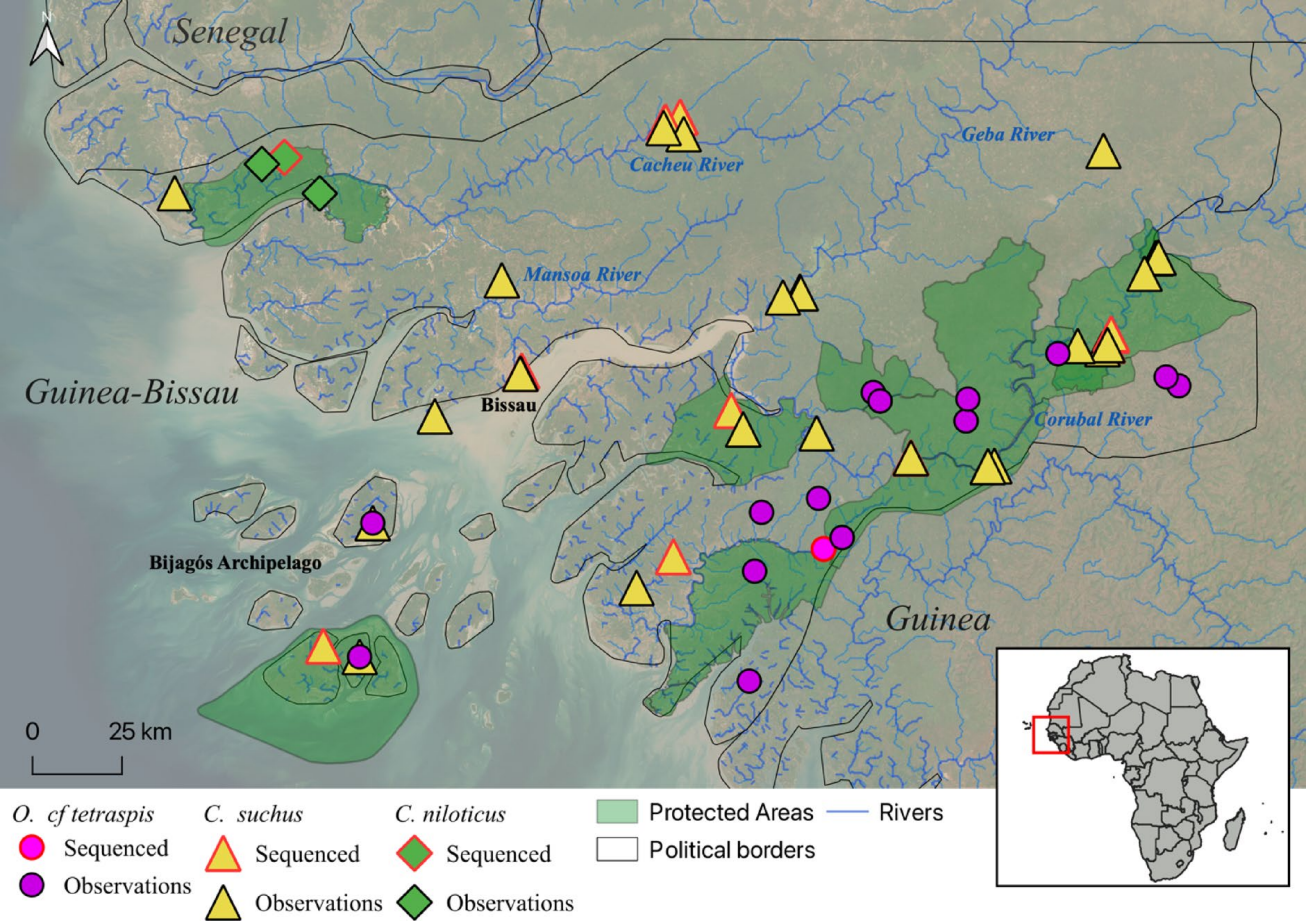
Distribution of crocodile data collected in Guinea-Bissau. Symbols with red strokes represent barcoded genetic samples.

We found molecular and phenotypic (aggressive behaviour towards humans and large size) evidence for the presence of *C. niloticus* at three sites in the western extension of the Cacheu River (Fig. 2). One of these *C. niloticus* observations was confirmed by the successful molecular barcoding of the two individuals killed in the 2015 incident. Other two sites were found in the western Cacheu River based on recorded non-fatal crocodile attacks of unusual large crocodiles. The most recent non-fatal crocodile attack we recorded in the region occurred on the 27th of January 2025 in the western Cacheu River, where a fisherman was bitten by an ambushing crocodile on the right leg while exiting the water.

We recorded *C. suchus* at 19 sites widely distributed across the country, mostly in the major rivers and tributaries and in fresh and brackish water lagoons. At 14 of these sites, we directly observed the species or found evidence of its presence (i.e., burrows, faeces and tracks), eight of which were confirmed by molecular identification. Two sites were found based on information from local people, and two were known from previous studies. The presence of juveniles and sub adults highlighted potentially reproducing populations in the western and eastern parts of the Cacheu River, the Geba River, the city of Bissau, the Bedasse and Cufada Lagoons, the Grande de Buba River, the Cumbidjã R. tributary, the Cacine River, and the Bijagós Islands.

We recorded *O. cf. tetraspis* at 15 sites distributed throughout southern Guinea-Bissau (Fig. 2). This species was found exclusively in small permanent or temporary rivers and pools surrounded by riparian forest, or in disturbed areas close to these sites (i.e., rice fields). We confirmed the presence of the species by direct observation of individuals in three sites, camera-traps records in eight sites, barcoding faecal samples in one site, and by bibliographical search in three sites.

### Conservation

We identified 13 types of threats potentially affecting crocodile populations and their habitats in 16 sites (Table 1). *Crocodylus suchus* was the species affected by more threats (12), followed by *O. cf. tetraspis* (9) and *C. niloticus* (5). Drought and persecution/control were the most recorded threats, followed by pollution from garbage, solid waste and soap. However, most of the threats recorded (10) have as consequence habitat loss and/or degradation. All the threats involving direct killing of crocodiles were classified as past, the six related to persecution/control were classified as likely to repeat, while the four related to war, and military exercises as unlikely to repeat.

**Table 1:** Conservation threats identified in this study for crocodiles in Guinea-Bissau. The table includes threat code, definition, species affected, number and percentage of sites where the threat has been observed (total number of sites = 19). Letters in the species column stand for: “n” *C. niloticus*, “s” *C. suchus*, “t” *Osteolaemus cf. tetraspis*. Coding and description are based on the IUCN red list classification scheme.

Code	Threats	Species	*N* of sites (%)
1.1	Housing & urban areas	n, s, t	3 (15.7)
1.2	Commercial & industrial areas	n, s, t	3 (15.7)
2.1.1	Shifting agriculture	n, s, t	4 (21)
2.3.1	Nomadic grazing	s, t	1 (5)
2.3.2	Small-holder grazing, ranching or farming	s, t	3 (15.7)
3.2	Mining & quarrying	s	1 (5)
5.4.3	Incidental or accidental mortality (bycatch - subsistence/small scale)	s, t	3 (15.7)
5.4.5	Persecution/control	n, s	6 (31.5)
6.2	War, civil unrest & military exercises	s, t	4 (21)
9.1.1	Sewage	s	2 (10.5)
9.1.3	Type Unknown/Unrecorded (Detergents)	s	5 (26.3)
9.4	Garbage & solid waste	s, t	6 (31.5)
11.2	Droughts	t	7 (36.8)

The two confirmed observations of *C. niloticus* come from two individuals killed by local authorities in 2015 because suspected of having eaten three people during 2015’s rainy season. Another possible *C. niloticus* (without molecular confirmation) was killed a few days later for the same reason at a second site in the Cacheu River. No retaliation events had followed the last human-crocodile incident that occurred on the 27th of January 2025 in the western Cacheu River. The four observations related to war, and military exercises come from the online resources, and were all recorded during the colonial period. Specifically, three are records of adults and sub-adults of either *C. niloticus* or *C. suchus* which were killed by the Portuguese military for bushmeat or during military operations between the years 1967 and 1974; one was a record of a *O. cf. tetraspis* also captured by the Portuguese military around 1973–1974^34^. Inquiry data indicated that, although crocodiles are occasionally eaten, they are not actively hunted for meat consumption, but their body parts are used in the production of goods (e.g., bags), traditional medicine and fetish rituals. One observation of *C. suchus* and one of *O. cf. tetraspis* were from individuals which accidentally died in fishing nets in the Cacheu River and in Belí (respectively).

Domestic garbage (e.g., single-use water plastic bags) is found throughout the country, but it is particularly abundant in urban and suburban areas. In these areas, streams, drainage channels and rice fields are filled with plastic and other degradable and non-degradable waste, from animal carcasses to discarded appliances. Pollution from soap was mainly observed in urban areas where people gather around large water bodies (e.g., in Saltinho, in the Corubal River or the mangrove forest in Bissau) to wash clothes, cars and themselves. Pollution from sewage was just recorded in Bissau.

We recorded evidence of land conversion, habitat degradation, and overexploitation of natural resources in the form of urban expansion, mining near major riverbanks, shifting (slash-and-burn) agriculture, and nomadic and non-nomadic grazing. Mining, and slash-and-burn agriculture were mainly recorded along the Corubal River, while nomadic and non-nomadic grazing was mostly recorded around freshwater lagoons (*wendus*). The intensifying land conversion recorded is particularly due to the increasing permanent habitat change from rainfed shifting agriculture to cashew orchards, as for instance along the Balana River.

Climate change was identified as a potential threat only to *O. cf. tetraspis*, as most of the remaining river pools where this species occurs may disappear if the duration of the dry season increases in the future^38^.

## Discussion

Our study gathers evidence of the presence of three crocodile species in Guinea-Bissau, distributed both on the mainland and in the Bijagós Archipelago. We have also highlighted the major threats to each species based on the IUCN Red List classification scheme, which are related to habitat loss, degradation, and direct killing of crocodiles.

### Species diversity and distribution

The molecular barcoding of samples collected from two crocodiles killed by local authorities in the Cacheu region during a culling operation in 2015, provides strong evidence on the presence of *C. niloticus* in West Africa, after the last confirmed observation 222 years ago^17^. These two crocodiles of 3.5 and 5 meters (full body length) were believed to be responsible for repeated attacks on pirogues and people during the 2015 rainy season, resulting in three fatalities and multiple injuries^34^. During our field expeditions surveys, we were also informed of the presence of large individuals (> 5 m) on the island of Imbone, in the Bijagós Archipelago (Miguel Lecoq personal observation). Human-crocodile conflicts were common in the Bijagós Archipelago in the past, often resulting in fatal encounters^35^. The attacks on humans and the large body size also suggest the presence of *C. niloticus* on the islands. This species was last confirmed in West Africa in 1803 in the Senegal River^17^. Until now, *C. niloticus* was thought extinct in the region, with *C. suchus* considered the sole species of the genus *Crocodylus*^17,20–22^. Due to the lack of an accepted dichotomous key to distinguish *C. niloticus* from *C. suchus* morphologically, attacks in the Cacheu region (and the few other attacks by crocodiles of the genus *Crocodylus* in West Africa) were attributed to *C. suchus* (https://crocattack.org/database/*).* However, our mitochondrial DNA analysis indicates that *C. niloticus* is likely responsible for the reported attacks on humans in the Cacheu region.

To date, no comprehensive public study has compared the phenotypic traits of these two species, making it urgent to investigate potential morphological and behavioural differences. Establishing clear dichotomous characters would improve field identification and aid conservation efforts. Although our morphological, behavioural, and genetic evidence strongly supports the presence of *C. niloticus* in the area, our analysis is based on a fragment of mtDNA. We cannot rule out the possibility that we are detecting *C. niloticus* mitochondrial introgression in *C. suchus*, which, if confirmed, would represent the first documented case of hybridization between the two species in the wild. However, it is important to note that karyotypes of *C. suchus* and *C. niloticus* differ in chromosomes number—34 and 32 chromosomes, respectively^17^ —which may present a reproductive barrier. Future research incorporating nuclear molecular markers is urgently needed to confirm the presence of *C. niloticus* populations in Guinea-Bissau and potentially in neighbouring countries such as the Republic of Guinea, where attacks have also been recorded (https://crocattack.org/database/). These areas should be promptly protected to ensure safe coexistence between human and crocodile populations.

Our results confirm the presence of *Osteolaemus tetraspis sensu lato* in Guinea-Bissau, both on the mainland and in the Bijagós Archipelago. Our molecular identification analyses place the barcoded samples within the same clade as the undescribed lineage *Osteolaemus cf. tetraspis* from West Africa. This clade has already been shown to be genetically divergent from the other two species of *Osteolaemus* (*O. tetraspis sensu stricto* and *O. osborni*)^26^. *Osteolaemus cf. tetraspis* was (and still is) widely confused with the neotropical species of caimans in Guinea-Bissau, and it is commonly referred to by the non-scientific community as “*jacaré*” (caiman in Brazilian Portuguese)^34^. Caimans (Caimaninae; Brochu, 1999) are small to medium-sized crocodilians of the family Alligatoridae Gray 1844, native to Central and South America. These species belong to the genera *Caiman*, *Melanosuchus*, and *Paleosuchus*, none of which have their natural distribution in Africa; therefore, no caiman (*jacaré*) species occur naturally in Guinea-Bissau.

Our observations of *O. cf. tetraspis* are restricted to southern Guinea-Bissau and the Bijagós Archipelago. Based on the available literature^27^ the distribution of *O. cf. tetraspis* should extend to northern Guinea-Bissau, south of Senegal and The Gambia. However, there are no recent observations of the species in southern Senegal (Casamance region), while the species was thought to be extinct in The Gambia prior to 2010^39^, when breeding populations were discovered^27^. The lack of updated observations and the increasing anthropization and land cover changes that have affected West Africa in recent decades^40–42^ may have restricted the current global distribution of *O.* cf. *tetraspis* to southern Guinea-Bissau and likely the Republic of Guinea, with possible isolated populations in The Gambia. There is an urgent need to survey the western limit of this species distribution and to assess whether *O. cf. tetraspis* still persists in The Gambia, southern Senegal and northern Guinea-Bissau, and to assess the status of these remaining populations.

During our field surveys, we found no direct evidence of the presence of *Mecistops cataphractus*. However, local hunters and fishermen consistently described sightings of a crocodile resembling *C. suchus* but with a brownish coloration and a longer, thinner head. These repeated accounts, along with numerous observations of unusually shaped crocodiles—especially in the south—raise intriguing questions about the possible presence of *M. cataphractus* or unrecognized morphological variations. Notably, the presence of this species in the region was previously highlighted^27^ further supporting the need for targeted investigations to clarify *M. cataphractus* presence in Guinea-Bissau.

### Conservation threats

Most of the threats recorded during our expeditions led to habitat loss and/or degradation. However, the most frequently observed were droughts and direct crocodile persecution. Although crocodiles are fully protected by CITES and Guinea-Bissau wildlife law^23^ crocodile body parts remain a valuable commodity in Guinea-Bissau. Although we do not have evidence that crocodile hunting still occurs in Guinea-Bissau few hunters actively hunt them using non-automatic firearms, spears or land traps (i.e. snares) until recently^35^. Although of uknown origin, *O. cf. tetraspis*, body parts were the most abundant crocodile items at the Bandim market in Bissau, the capital city, even surpassing those of *C. suchus* (CP personal observation). Due to its small size and tame behaviour, *O. cf. tetraspis* is highly traded throughout its distribution^31,32,43^. Overhunting can lead to extinction, and disrupt population dynamics and activity patterns^44,45^ while unregulated bushmeat trade and consumption can increase the risk of zoonotic diseases^46,47^. However, little is still known on the extent of crocodile trade in Guinea-Bissau, nor on the socio-economic drivers behind the trade in crocodile body parts. Additionally, the lack of information on population size, distribution, and trends—both current and historical—combined with the uncertainty surrounding the scale of the trade, poses a serious threat to the species’ persistence in the country. Urgent studies are needed to fill these knowledge gaps, strengthen conservation efforts, and prevent the silent disappearance of this still-undescribed species from Guinea-Bissau.

The direct killing of crocodiles in Guinea-Bissau has mostly been driven by persecution due to human-crocodile conflict and, historically, by warfare. Crocodile hunting in Guinea-Bissau was intense during the colonial period, including with the use of automatic machine guns and grenades, and was often carried out by the Portuguese military, who had easy access to firearms^34^. Human-wildlife conflicts can be driven by several factors including climate change^48^ and the intensification of environmental stressors, such as land conversion, habitat degradation, and overexploitation of natural resources^49–51^. The human population of Guinea-Bissau has grown by approximately 120% from 1990 to 2024^52^, intensifying environmental pressure. This has led to habitat loss due to urban expansion and commodity crop cultivation^53^ as well as habitat degradation caused by chemical pollution (e.g., domestic detergents) and slash-and-burn agriculture^54^. These changes likely exacerbate the risk of human-crocodile conflict and can result in direct disturbance and reduction of reproductive opportunities, for example through the destruction of nesting and nursery habitats (i.e. sandbanks and mangroves). The use of fire in slash-and-burn agriculture to clear natural patches of riparian forest along the Corubal River is another example. The degradation and depletion of riparian forests may lead to the rapid extinction of *O. cf. tetraspis*^43^ which species depends on the persistence of these forested environments^55^. The increase in nomadic and non-nomadic grazing, particularly around freshwater lagoons (*wendus*), also poses a threat to the persistence of crocodiles as it may lead to water shortage and pollution^56^. Finally, if the length of the dry season increases as predicted^38^ suitable habitat for *O. cf. tetraspis* might also disappear in the near future. Holistic approaches, such as agroecology, could be valuable in addressing human-wildlife conflict while implementing sustainable farming and wildlife management^57^. There is an urgent need to strengthen national and sub-national policies on habitat protection and pollution control, alongside implementing projects that promote habitat restoration and raise awareness of the importance of conserving natural ecosystems. These efforts will be crucial to mitigating the possible negative impacts of advancing climate change.

## Conservation management and future research

Based on our findings, there is a critical need for comprehensive conservation management and research to address the lack of knowledge on crocodiles in Guinea-Bissau and the threats they are facing. As unassessed biodiversity is more likely to go extinct^58^ the taxonomic clarification of the genus *Osteolaemus* is urgently needed to inform and guide conservation efforts for the species in Guinea-Bissau and neighbouring countries. Future research should focus on standardized and systematic monitoring of known crocodile populations, their habitats, and trade trends, all of which are essential for more effective conservation measures. Moreover, future research should focus on the codification of dichotomous morphological characters to distinguish *C. suchus* and *C. niloticus*, as well as the use of molecular methods to confirm species identification and investigate the presence of *C. niloticus* in surrounding regions where human-crocodile conflicts are also occurring (e.g., Republic of Guinea). Additionally, the presence of *M. cataphractus* in Guinea-Bissau remains uncertain, despite reports from local hunters and fishermen describing crocodiles with distinct morphological traits. Given these accounts and previous indications^27^ further research is needed to clarify crocodile diversity in the region.

Conservation actions should focus on site and habitat protection, area management, and restoration of natural processes that have been lost, especially in areas critical for the survival of *O. cf. tetraspis* (i.e., urban areas in the northwest of the country), and to facilitate the coexistence of the human population with the potentially vulnerable populations of *C. niloticus*. Maintaining the protection of sacred forests has proved to be important for crocodiles^59^. Although shifts in religious beliefs in Guinea-Bissau are affecting traditional practices and the preservation of sacred forests, community-managed forests are increasingly valued in the country^60^ offering a promising solution for local crocodile conservation. Initiatives like Fédération KAFO in Djalicunda (https://kafobissau.org/en/) and CHIMBO in Belí (https://www.chimbo.org) promote wildlife conservation through research and sustainable agroecological practices, preserving natural and cultural heritage, protecting climate-resilient ecosystems, and empowering young people to safeguard the environment. Combining community-based approaches with management strategies based on genetic research and trade regulation is essential to preserve genetic diversity and mitigate exploitation risks. Enhancing education, training, and community awareness will further reduce human-crocodile conflicts and promote crocodile conservation through biodiversity-friendly practices.

## Materials and methods

### Study area

**Fig. 3:**
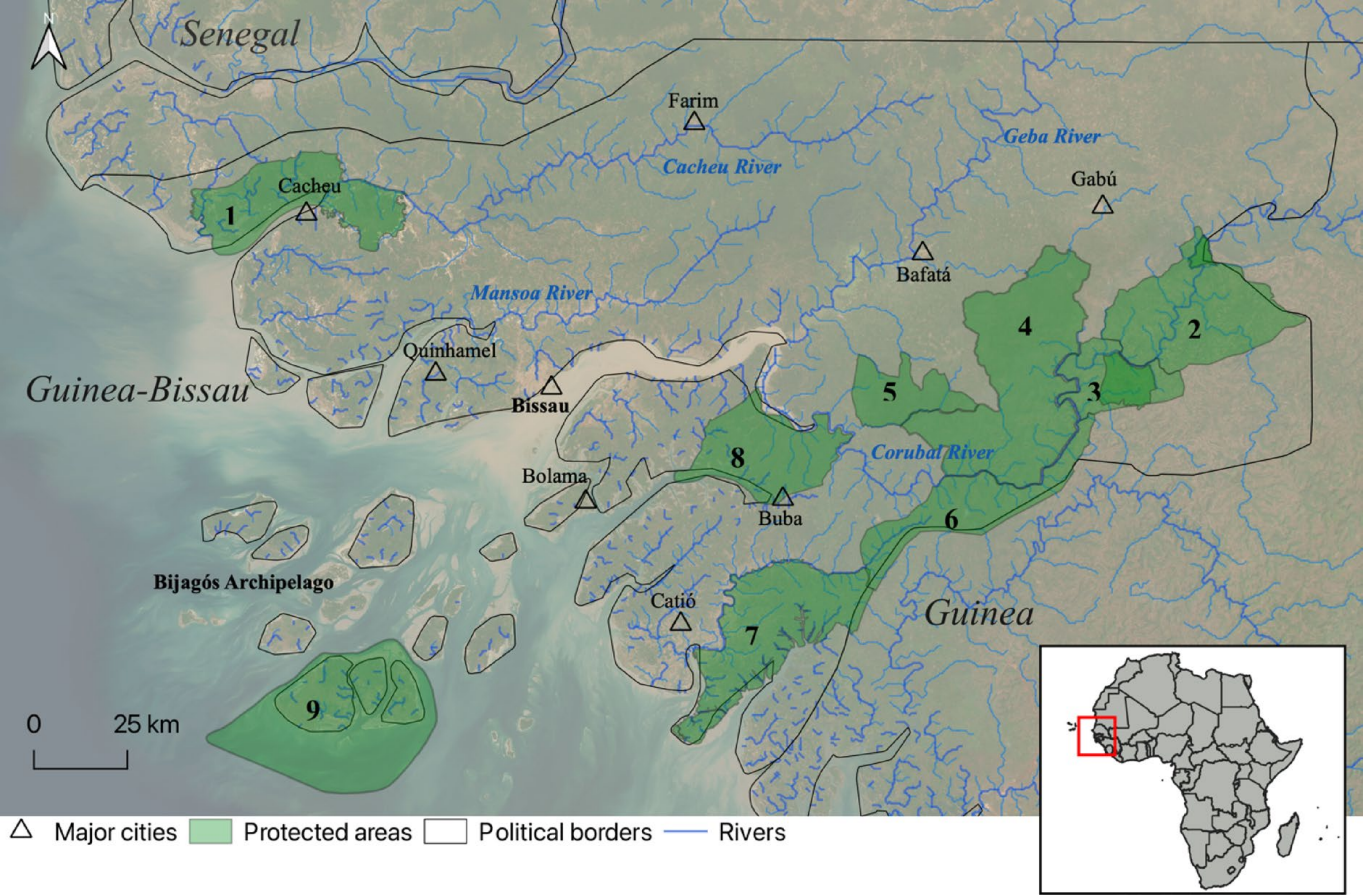
Map of the toponymies in the study area. Triangles indicate the major cities, whose names are adjacent to them. Numbers in bold indicate the protected areas: (1) Tarrafes do Rio Cacheu National Park, (2) Boe National Park, (3) Tchetche Natural Corridor, (4) Dulombi National Park, (5) Salifo-Xitole Natural Corridor, (6) Cuntabani-Quebo Natural Corridor, (7) Cantanhez National Park, (8) Lagoas de Cufada Natural Park and (9) Grupo de Ilhas de Orango National Park. The red rectangle in the inset map of Africa represents the geographic location of Guinea-Bissau. This figure and maps within it, were produced using QGIS version 3.36 (QGIS Development Team, 2024; https://qgis.org).

Guinea-Bissau, located in West Africa, is bordered by Senegal, the Republic of Guinea, and the Atlantic Ocean (Fig. 3). It covers 36,125 km², with a population of about 2.1 million^52^. The country features two main ecoregions: the Guinean forest-savannah mosaic and Guinean mangroves. The landscape includes scattered coastal plains, estuaries lined with mangroves, a low-elevation mosaic of dense and open forests, and savannah woodland. The Bijagós Archipelago comprises 88 islands, 20 of which are permanently inhabited. The monsoonal tropical climate has a dry season (November–May) and a wet season (June–October), with annual rainfall ranging from 1,500 to 3,000 mm. Vegetation includes mangroves, palm groves, wet grasslands, dry open forest, savanna woodland, dense primary and secondary forest in relict patches, and riverine galleries. The main river basins in the country are the Cacheu in the north, the Mansoa and the Geba in the centre, and the Corubal in the south (Fig. 3).

### Data collection

We visited the country during three expeditions, between 2021 and 2023. The expeditions were conducted in November/December 2021 (dry season), October/November 2022 (late rainy season, early dry season), and late April/May 2023 (dry season), for a total of 74 survey-days. During these expeditions we visited the mainland’s protected areas and most of the main rivers: Cacheu, Corubal, Geba, and Mansoa, as well as smaller tributaries and temporary rainfed lagoons (locally known as *wendus*) (Fig. 3).

Data were collected by: (1) visual surveys; (2) opportunistic sampling of genetic material (faeces and tissues) along rivers, lagoons, and other water bodies; (3) opportunistic bycatch camera-trapping data from eight sites in the south of the country, from a project aimed to survey forest elephants from 2020 to 2024^61^; (4) opportunistic presence data from interviewing knowledgeable local people (i.e. natural guides, park rangers, hunters, shepherds, and fishermen); (5) occasional visits to bushmeat and fetish markets in Bissau where we counted the number of stalls displaying items made from crocodile body parts; (6) bibliographic review of the available scientific and non-scientific literature. We reached survey and sampling sites by car and where possible, by canoe, accompanied by local guides, park rangers or fishermen. During boat trips, we counted individuals and collected evidence of crocodile presence (i.e. faeces, tracks and burrows). We conducted day and night visual surveys from canoes. During the canoe surveys we navigated along water bodies, with two to four people spotting live individuals or evidence of crocodile presence. Where navigation was not possible (e.g. small pools along temporary rivers under riparian forest), we reached the water bodies by car or on foot and then walked along and inside them at random. Tissue samples were collected from dead individuals killed by humans. Faecal samples were collected using sterile, disposable throat inspection wooden sticks. Tissue samples were stored in 96% ethanol until DNA extraction, while faecal samples were stored in 50 ml Falcon tubes filled with sterile silica gel. The geographic location of each observation or genetic sample was recorded using a Global Positioning System (GPS) device. All methods were carried out in accordance with relevant guidelines and regulations. Fieldwork and data collection were conducted with permission from IBAP. Interviews were informal, unstructured, and anonymous, and responses were kept confidential. Informed consent was obtained from all participants involved in the study.

We also recorded threats that may affect crocodiles in the country and classified them based on the Unified Classification of Direct Threats Version 3.3 provided by the IUCN Red List^62^.

### Molecular identification

For DNA extraction, we followed^20^. We extracted DNA from tissues samples using the DNeasy Blood & Tissue Kit (QIAGEN) and from faecal samples using the GuSCN/silica method^63^. For faecal samples we performed extraction and pre-PCR procedures at BIOPOLIS-CIBIO research centre in dedicated low-quality DNA facilities, sterilised and equipped with positive air pressure and UV lights.

For DNA barcoding analyses we amplified a 421 bp (bp) 12S mitochondrial DNA (mtDNA) fragment in 10 µl reaction volume, containing 5 µl of master mix (Multiplex PCR Kit, QIAGEN), 0.5 μm of each primer and between 1 and 2 µl of DNA for tissue and faecal samples, respectively. Primer information and PCR conditions were the same as in Table S2 of Velo-Anton et al. (2014). We performed cycle sequencing for both strands using PCR primers and the ABI PRISM BigDye Terminator kit (AB Applied Biosystems) in a MyCycler BioRad Thermal Cycler and sequenced PCR products on an ABI 3130xl Genetic Analyzer (AB Applied Biosystems).

We checked and edited electropherograms by eye and aligned all sequences using GENEIOUS PRIME 2024.0.7. We then downloaded available 12S sequences of *Crocodylus niloticus*,* C. suchus*, *C. porosus* (Schneider, 1801), *Osteolaemus osborni*,* O. tetraspis*,* O. cf. tetraspis*,* Mecistops cataphractus*,* M. leptorhynchus*,* Gavialis gangeticus* (Gmelin, 1789), *Tomistoma schlegelii* (Müller, 1838) from previous publications^20,26,37,64–66^.

The final alignment contains a total of 142 sequences with a length of 313 bp. The first step of our species identification was performed in BLASTn (https://blast.ncbi.nlm.nih.gov/Blast.cgi*)* to compare our query sequences with the NCBI reference sequence database. Sequences were identified at the species level when the percentage of identification was higher than 96% for the first 10 matching sequences in the NCBI database. Since some available sequences from *C. suchus* are still named as *C. niloticus* in the NCBI database, we performed a Bayesian inference analysis using BEAST 2.5^67^ to establish the correct phylogenetic position of our sequences from the three genera. We run three separate analyses, each for 10^7^ generations with sampling at every 10^3^ MCMC steps. We used the strict clock model and the Coalescent Constant Population prior. We used IQ-TREE^68^ to infer the model to be assigned to the appropriate partitions. We used Tracer V1.7.2^69^ to visualise the log file of each run and summarise the analytic statistics. The effective sample size was sufficient for all statistics (ESS > 200). We then used LogCombiner to combine the tree files from each run discarding a 10% burn-in. We used TreeAnnotator to produce the maximum credibility tree and annotate the 95% highest probability density ranges.

## Electronic supplementary material

Below is the link to the electronic supplementary material.

Supplementary Material 1

## Data Availability

The datasets generated and analysed during the current study are available in the Zenodo repository at https://doi.org/10.5281/zenodo.14975165.
