# Supplementary material for: Diversity, distribution and conservation of crocodiles (Order: Crocodylia) in Guinea-Bissau, West Africa
Source: Sci Rep. 2025 Jul 9;15:24703. doi: 10.1038/s41598-025-08789-3 (PMC12241385; doi:10.1038/s41598-025-08789-3)
Supplement: Supplementary file 1 — Supplementary Material 1 [file 41598_2025_8789_MOESM1_ESM.docx]

**Supplementary information**

**Diversity, distribution and conservation of crocodiles (Order: Crocodylia) in Guinea-Bissau, West Africa**

Cristian Pizzigalli^1,2,3,4^*, Aissa Regalla^5^, Ana Filipa Palmeirim^1,2,6^, Luís Palma^1,2,7^, Manuel Lopes-Lima^1,2,3^, Orly Razgour^4^, Raquel Godinho^1,2,3,8^, William Alexandre Intipe^5^, and José Carlos Brito^1,2,3^

1 CIBIO, Centro de Investigação em Biodiversidade e Recursos Genéticos, Campus de Vairão, Universidade do Porto, 4485-661 Vairão, Portugal

2 BIOPOLIS Program in Genomics, Biodiversity and Land Planning, CIBIO, Campus de Vairão, 4485-661 Vairão, Portugal

3 Departamento de Biologia da Faculdade de Ciências, Universidade do Porto, Porto, Portugal

4 Biosciences, Faculty of Health and Life Sciences, University of Exeter, Exeter, UK

5 Instituto da Biodiversidade e das Áreas Protegidas (IBAP), [VCF4+84M, Bissau, Guinea-Bissau](https://www.google.com/maps/place//data=!4m2!3m1!1s0xee6db035714847d:0x21319a8644cd4d4c?sa=X&ved=1t:8290&ictx=111)

6 Laboratório de Ecologia e Zoologia de Vertebrados, Instituto de Ciências Biológicas, Universidade Federal do Pará, Brasil

7 CIBIO, Centro de Investigação em Biodiversidade e Recursos Genéticos, Instituto Superior de Agronomia, Universidade de Lisboa, 1349-017 Lisboa, Portugal

8 Department of Zoology, Faculty of Sciences, University of Johannesburg, Auckland Park 2006, South Africa

**Table S1.** The list and information of the samples collected for this study.

| **Code** | **Species** | **Sequenced** | **Year** | **Province** | **Sample type** |
| --- | --- | --- | --- | --- | --- |
| B8 | *Crocodylus suchus* |  | 1968 | Biombo | Bibliography |
| B6 | *Crocodylus suchus* |  | 1969 | Tombali | Bibliography |
| B9 | *Crocodylus suchus* |  | 1971 | Bafatá | Bibliography |
| B7 | *Crocodylus suchus* |  | 1973 | Cacheu | Bibliography |
| B5 | *Osteolaemus cf tetraspis* |  | 1975 | Tombali | Bibliography |
| ff - 5323 | *Crocodylus suchus* | yes | 2010 | Bolama | Tissue |
| B1 | *Crocodylus suchus* |  | 2012 | Formosa | Bibliography |
| B2 | *Osteolaemus cf tetraspis* |  | 2012 | Formosa | Bibliography |
| B3 | *Crocodylus suchus* |  | 2012 | Orango | Bibliography |
| B4 | *Osteolaemus cf tetraspis* |  | 2012 | Orango | Bibliography |
| 12915 | *Crocodylus niloticus* |  | 2015 | Cacheu | Direct observation |
| 13263 | *Crocodylus niloticus* | yes | 2015 | Cacheu | Tissue |
| 13264 | *Crocodylus niloticus* | yes | 2015 | Cacheu | Tissue |
| B10 | *Crocodylus suchus* |  | 2020 | Bissora | Bibliography |
| 19648 | *Crocodylus suchus* |  | 2021 | Bissau | Inquiry |
| 19926 | *Crocodylus suchus* |  | 2021 | Quinara | Faeces |
| 19927 | *Crocodylus suchus* | yes | 2021 | Quinara | Faeces |
| 19928 | *Crocodylus suchus* | yes | 2021 | Bissau | Faeces |
| 19929 | *Crocodylus suchus* | yes | 2021 | Bissau | Faeces |
| 19930 | *Crocodylus suchus* |  | 2021 | Bafata | Direct observation |
| 19934 | *Crocodylus suchus* | yes | 2021 | Tombali | Faeces |
| 19935 | *Osteolaemus cf tetraspis* |  | 2021 | Tombali | Direct observation |
| 19936 | *Osteolaemus cf tetraspis* |  | 2021 | Tombali | Direct observation |
| 19961 | *Crocodylus suchus* |  | 2021 | Bafata | Faeces |
| 19962 | *Crocodylus suchus* |  | 2021 | Bafata | Faeces |
| 19970 | *Crocodylus suchus* |  | 2021 | Bissau | Faeces |
| 19971 | *Crocodylus suchus* | yes | 2021 | Bissau | Faeces |
| 19972 | *Crocodylus suchus* |  | 2021 | Bissau | Faeces |
| 19973 | *Crocodylus suchus* |  | 2021 | Bissau | Faeces |
| 19974 | *Crocodylus suchus* | yes | 2021 | Bissau | Faeces |
| 19975 | *Crocodylus suchus* |  | 2021 | Bissau | Faeces |
| 19982 | *Crocodylus suchus* |  | 2021 | Bafata | Direct observation |
| 19999 | *Crocodylus suchus* |  | 2021 | Bissau | Direct observation |
| 20000 | *Crocodylus suchus* |  | 2021 | Gabú | Inquiry |
| 20001 | *Crocodylus suchus* |  | 2021 | Bissau | Direct observation |
| 20002 | *Osteolaemus cf tetraspis* |  | 2021 | Tombali | Direct observation |
| CP49 | *Osteolaemus cf tetraspis* |  | 2021 | Gabu | Direct observation |
| CP50 | *Osteolaemus cf tetraspis* |  | 2021 | Gabu | Direct observation |
| 20456 | *Crocodylus suchus* | yes | 2022 | Gabu | Faeces |
| 20457 | *Crocodylus suchus* |  | 2022 | Bafatá | Direct observation |
| 20693 | *Crocodylus suchus* |  | 2022 | Oio | Faeces |
| 20718 | *Crocodylus suchus* | yes | 2022 | Oio | Faeces |
| 20719 | *Crocodylus suchus* | yes | 2022 | Oio | Faeces |
| 20720 | *Crocodylus suchus* | yes | 2022 | Oio | Faeces |
| 20725 | *Crocodylus suchus* | yes | 2022 | Oio | Faeces |
| 20728 | *Crocodylus suchus* | yes | 2022 | Bissau | Faeces |
| 20729 | *Crocodylus suchus* | yes | 2022 | Bissau | Faeces |
| CP10 | *Crocodylus suchus* |  | 2022 | Oio | Direct observation |
| CP11 | *Crocodylus suchus* |  | 2022 | Oio | Direct observation |
| CP12 | *Crocodylus suchus* |  | 2022 | Oio | Direct observation |
| CP13 | *Crocodylus suchus* |  | 2022 | Oio | Direct observation |
| CP14 | *Crocodylus suchus* |  | 2022 | Oio | Direct observation |
| CP15 | *Crocodylus suchus* |  | 2022 | Oio | Direct observation |
| CP16 | *Crocodylus suchus* |  | 2022 | Oio | Direct observation |
| CP17 | *Crocodylus suchus* |  | 2022 | Oio | Direct observation |
| CP18 | *Crocodylus suchus* |  | 2022 | Oio | Direct observation |
| CP2 | *Crocodylus suchus* |  | 2022 | Oio | Direct observation |
| CP21 | *Crocodylus suchus* |  | 2022 | Oio | Direct observation |
| CP22 | *Crocodylus suchus* |  | 2022 | Oio | Direct observation |
| CP23 | *Crocodylus suchus* |  | 2022 | Oio | Direct observation |
| CP25 | *Crocodylus suchus* |  | 2022 | Oio | Direct observation |
| CP26 | *Crocodylus suchus* |  | 2022 | Oio | Direct observation |
| CP27 | *Crocodylus suchus* |  | 2022 | Oio | Direct observation |
| CP28 | *Crocodylus suchus* |  | 2022 | Oio | Direct observation |
| CP29 | *Crocodylus suchus* |  | 2022 | Oio | Direct observation |
| CP3 | *Crocodylus suchus* |  | 2022 | Oio | Direct observation |
| CP30 | *Crocodylus suchus* |  | 2022 | Oio | Direct observation |
| CP31 | *Crocodylus suchus* |  | 2022 | Oio | Direct observation |
| CP35 | *Crocodylus suchus* |  | 2022 | Oio | Direct observation |
| CP4 | *Crocodylus suchus* |  | 2022 | Oio | Direct observation |
| CP5 | *Crocodylus suchus* |  | 2022 | Oio | Direct observation |
| CP6 | *Crocodylus suchus* |  | 2022 | Oio | Direct observation |
| CP7 | *Crocodylus suchus* |  | 2022 | Oio | Direct observation |
| CP8 | *Crocodylus suchus* |  | 2022 | Oio | Direct observation |
| CP9 | *Crocodylus suchus* |  | 2022 | Oio | Direct observation |
| L1 | *Osteolaemus cf tetraspis* |  | 2022 | Boé | Camera-trap |
| L2 | *Osteolaemus cf tetraspis* |  | 2022 | Boé | Camera-trap |
| 20780 | *Crocodylus suchus* |  | 2023 | Gabu | Faeces |
| 20781 | *Crocodylus suchus* |  | 2023 | Gabu | Direct observation |
| 20782 | *Crocodylus suchus* | yes | 2023 | Gabu | Faeces |
| 20783 | *Crocodylus suchus* | yes | 2023 | Gabu | Faeces |
| 20784 | *Crocodylus suchus* |  | 2023 | Gabu | Faeces |
| 20785 | *Crocodylus suchus* | yes | 2023 | Gabu | Faeces |
| 20786 | *Crocodylus suchus* | yes | 2023 | Gabu | Faeces |
| 20787 | *Crocodylus suchus* | yes | 2023 | Gabu | Faeces |
| 20788 | *Crocodylus suchus* | yes | 2023 | Gabu | Faeces |
| 20789 | *Crocodylus suchus* | yes | 2023 | Gabu | Faeces |
| 20790 | *Crocodylus suchus* | yes | 2023 | Gabu | Faeces |
| 20791 | *Crocodylus suchus* | yes | 2023 | Gabu | Faeces |
| 20792 | *Crocodylus suchus* |  | 2023 | Gabu | Tracks |
| 20793 | *Crocodylus suchus* | yes | 2023 | Gabu | Faeces |
| 20794 | *Crocodylus suchus* |  | 2023 | Gabu | Faeces |
| 20795 | *Crocodylus suchus* | yes | 2023 | Gabu | Faeces |
| 20796 | *Crocodylus suchus* | yes | 2023 | Gabu | Faeces |
| 20797 | *Crocodylus suchus* | yes | 2023 | Gabu | Faeces |
| 20798 | *Crocodylus suchus* |  | 2023 | Gabu | Faeces |
| 20799 | *Crocodylus suchus* | yes | 2023 | Gabu | Faeces |
| 20800 | *Crocodylus suchus* | yes | 2023 | Gabu | Faeces |
| 20801 | *Crocodylus suchus* | yes | 2023 | Gabu | Faeces |
| 20802 | *Crocodylus suchus* | yes | 2023 | Gabu | Faeces |
| 20803 | *Crocodylus suchus* | yes | 2023 | Gabu | Faeces |
| 20804 | *Crocodylus suchus* | yes | 2023 | Gabu | Faeces |
| 20805 | *Crocodylus suchus* | yes | 2023 | Gabu | Faeces |
| 20806 | *Crocodylus suchus* | yes | 2023 | Gabu | Faeces |
| 20807 | *Crocodylus suchus* | yes | 2023 | Gabu | Faeces |
| 20808 | *Crocodylus suchus* | yes | 2023 | Gabu | Faeces |
| 20809 | *Crocodylus suchus* |  | 2023 | Gabu | Tracks |
| 20810 | *Crocodylus suchus* |  | 2023 | Gabu | Faeces |
| 20811 | *Crocodylus suchus* |  | 2023 | Gabu | Tracks |
| 20812 | *Crocodylus suchus* |  | 2023 | Gabu | Inquiry |
| 20813 | *Crocodylus suchus* |  | 2023 | Gabu | Tracks |
| 20814 | *Osteolaemus cf tetraspis* | yes | 2023 | Tombali | Faeces |
| 20815 | *Osteolaemus cf tetraspis* | yes | 2023 | Tombali | Faeces |
| 20816 | *Osteolaemus cf tetraspis* | yes | 2023 | Tombali | Faeces |
| 20817 | *Crocodylus suchus* |  | 2023 | Gabu | Faeces |
| 20818 | *Crocodylus suchus* |  | 2023 | Gabu | Faeces |
| 20819 | *Crocodylus suchus* |  | 2023 | Quinar | Direct observation |
| 20820 | *Crocodylus suchus* | yes | 2023 | Bafata | Faeces |
| 20821 | *Crocodylus suchus* | yes | 2023 | Bafata | Faeces |
| 20822 | *Crocodylus suchus* | yes | 2023 | Bafata | Faeces |
| 20823 | *Crocodylus suchus* | yes | 2023 | Bafata | Faeces |
| 20824 | *Crocodylus suchus* | yes | 2023 | Bafata | Faeces |
| 20825 | *Crocodylus suchus* | yes | 2023 | Bafata | Faeces |
| 20826 | *Crocodylus suchus* | yes | 2023 | Bafata | Faeces |
| 20827 | *Crocodylus suchus* | yes | 2023 | Bafata | Faeces |
| 20828 | *Crocodylus suchus* | yes | 2023 | Bafata | Faeces |
| 20829 | *Crocodylus suchus* |  | 2023 | Bafata | Faeces |
| 20830 | *Crocodylus suchus* | yes | 2023 | Bafata | Faeces |
| 20831 | *Crocodylus suchus* |  | 2023 | Bafata | Faeces |
| 20832 | *Crocodylus niloticus* |  | 2023 | Orango Island | Inquiry |
| CP36 | *Crocodylus suchus* |  | 2023 | Oio | Direct observation |
| CP39 | *Crocodylus suchus* |  | 2023 | Oio | Direct observation |
| CP44 | *Crocodylus suchus* |  | 2023 | Tombali | Direct observation |
| CP46 | *Crocodylus suchus* |  | 2023 | Quinar | Direct observation |
| CP48 | *Crocodylus suchus* |  | 2023 | Bissau | Direct observation |
| L10 | *Osteolaemus cf tetraspis* |  | 2023 | Gabu | Camera-trap |
| L11 | *Osteolaemus cf tetraspis* |  | 2023 | Gabu | Camera-trap |
| L13 | *Osteolaemus cf tetraspis* |  | 2023 | Gabu | Camera-trap |
| L14 | *Osteolaemus cf tetraspis* |  | 2023 | Quebo | Camera-trap |
| L15 | *Osteolaemus cf tetraspis* |  | 2023 | Quebo | Camera-trap |
| L16 | *Osteolaemus cf tetraspis* |  | 2023 | Quebo | Camera-trap |
| L17 | *Osteolaemus cf tetraspis* |  | 2023 | Quebo | Camera-trap |
| L18 | *Osteolaemus cf tetraspis* |  | 2023 | Quebo | Camera-trap |
| L19 | *Osteolaemus cf tetraspis* |  | 2023 | Quebo | Camera-trap |
| L20 | *Osteolaemus cf tetraspis* |  | 2023 | Quebo | Camera-trap |
| L6 | *Osteolaemus cf tetraspis* |  | 2023 | Gabu | Camera-trap |
| L7 | *Osteolaemus cf tetraspis* |  | 2023 | Gabu | Camera-trap |
| L8 | *Osteolaemus cf tetraspis* |  | 2023 | Gabu | Camera-trap |
| L9 | *Osteolaemus cf tetraspis* |  | 2023 | Gabu | Camera-trap |
| L21 | *Osteolaemus cf tetraspis* |  | 2024 | Xitole | Camera-trap |
| L22 | *Osteolaemus cf tetraspis* |  | 2024 | Xitole | Camera-trap |
| L23 | *Osteolaemus cf tetraspis* |  | 2024 | Xitole | Camera-trap |
| L24 | *Osteolaemus cf tetraspis* |  | 2024 | Xitole | Camera-trap |
| L25 | *Osteolaemus cf tetraspis* |  | 2024 | Xitole | Camera-trap |
| L26 | *Osteolaemus cf tetraspis* |  | 2024 | Xitole | Camera-trap |
| L27 | *Osteolaemus cf tetraspis* |  | 2024 | Xitole | Camera-trap |
| L28 | *Osteolaemus cf tetraspis* |  | 2024 | Xitole | Camera-trap |
| W1 | *Crocodylus niloticus* |  | 2025 | Cacheu | Inquiry |
